# Ubiquitylation as a Rheostat for TCR Signaling: From Targeted Approaches Toward Global Profiling

**DOI:** 10.3389/fimmu.2015.00618

**Published:** 2015-12-16

**Authors:** Claire E. O’Leary, Emma L. Lewis, Paula M. Oliver

**Affiliations:** ^1^Perelman School of Medicine, University of Pennsylvania, Philadelphia, PA, USA

**Keywords:** ubiquitin, TCR signaling, proteomics, ubiquitin-protein ligases, T cell

## Abstract

T cell receptor (TCR) signaling must be precisely tuned to limit collateral damage and prevent reactivity to self, while still allowing robust protective immune responses that control pathogen invasion. One process that can be used to promote, modify, or terminate TCR signaling is ubiquitylation. During ubiquitylation, ubiquitin is covalently attached to target proteins through a multistep process, in which E3 ubiquitin ligases promote the formation of ubiquitin chains on selected substrates. Ubiquitylation can facilitate protein–protein interactions, direct a protein to a specific subcellular location, or initiate protein destruction. Like phosphorylation, ubiquitylation is a reversible process – deubiquitylating enzymes counteract ligase function by removing ubiquitin chains. This reversibility also allows for ubiquitin chain “editing.” Based on an emerging wealth of information from genetic loss-of-function studies showing that deregulation of ubiquitylation pathways leads to immune dysfunction, it has become increasingly apparent that the dynamic process of ubiquitylation is critical for normal immune cell function. In this review, we will describe how ubiquitylation acts as a key modulator and integrator of signaling downstream of TCR engagement. Specifically, we highlight the known roles of the substrate-specific E3 ligases and deubiquitylating enzymes in TCR signaling and T cell activation. While it is clear that ubiquitin enzymes tune T cell signaling and T cell function, elucidating the molecular mechanisms by which these proteins modulate T cells has met with significant challenges. Identifying substrates of these enzymes has been a particular challenge, and thus substrates of many E3 ligases and deubiquitylating enzymes remain largely unknown. To that end, we discuss the promise, and some practical considerations, of using proteomics-based techniques for unbiased identification of putative substrates of ubiquitin cascade proteins within primary T cells. These methods provide an exciting opportunity for further defining how TCR signals are regulated and for identifying new targets for therapeutic modulation.

## Introduction

T cells are key organizers of the immune response. During an immune response, CD4^+^ T helper cells direct the response of other cell types through the production of cytokines, while CD8^+^ cytotoxic T cells mediate direct elimination of infected or altered-self cells. Both CD4^+^ and CD8^+^ T cells promote elimination of pathogens through a variety of means and provide protection against re-exposure by establishing long-lasting memory cell populations. To acquire these protective functions, T cells are first activated via their T cell receptor (TCR) by an antigen-presenting cell (APC) presenting an antigenic peptide-MHC (pMHC). During activation, quantitative (strength) as well as qualitative (presence or absence) signals are integrated to specifically tailor the T cell response to the host’s need to clear a specific type of pathogen or limit collateral damage from other responding immune cells. The combination of signals from engagement of the TCR, co-stimulatory molecules, and cytokine receptors (also known as, respectively, signals one, two, and three) allows appropriate, contextual activation and differentiation of T cells (1–4). Depending on the type of pathogen encountered, CD8^+^ T cells and distinct T helper subsets play more or less important roles in directing the immune response.

Inappropriate signaling downstream of TCR/co-stimulatory molecule engagement can lead a T cell to respond aberrantly, resulting in hypo- or hyper-responsiveness to antigenic stimulation (5–7). The expression levels and function of cellular proteins that relay signals initiated downstream of TCR and co-stimulatory molecule engagement are therefore tightly regulated to prevent inappropriate T cell responses. Post-translational modifications play critical roles in regulating these signal transduction pathways. In this regard, phosphorylation and dephosphorylation events have been a primary focus of research on TCR signaling pathways, particularly TCR-proximal signaling. However, there is growing appreciation that covalent attachment of the 8-kDa protein ubiquitin, either single or in chains, is another key post-translational modification driving T cell signaling and fate decisions.

The most commonly considered outcome of ubiquitylation is protein degradation via the proteasome. However, monoubiquitylation or polyubiquitylation of accessible target protein lysines can alter protein fate and function in diverse ways, resulting in protein degradation, activation or inhibition of function, altered trafficking, or providing a scaffold for protein–protein interactions (8, 9). In T cells, ubiquitylation can affect T cell activation and anergy, as well as helper T cell differentiation, cytokine production, and cell cycle progression (10, 11).

**Figure 1:**
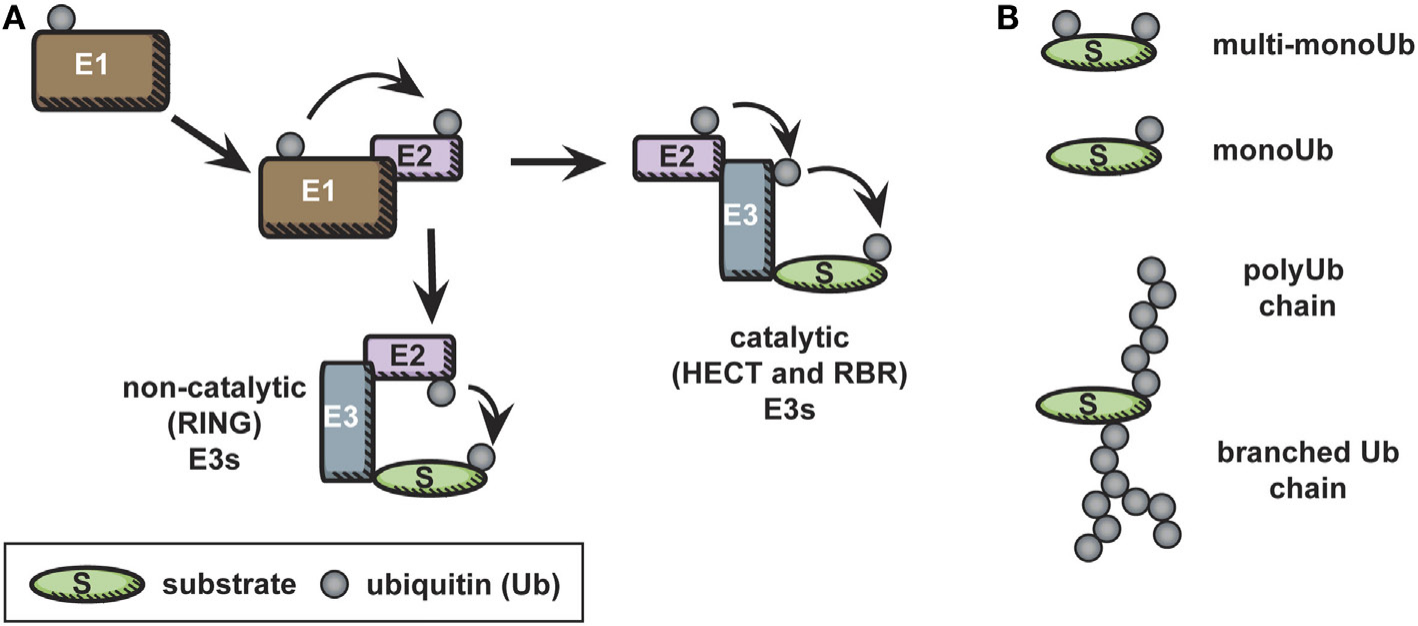
**The ubiquitin cascade**. **(A)** Free ubiquitin is activated in an ATP-dependent manner by conjugation to an E1. Subsequently, in a trans-thiolation reaction, ubiquitin is transferred from the E1 to the catalytic cysteine of an E2. The ubiquitin-conjugated E2 then interacts with an E3 ubiquitin ligase, to allow ubiquitylation on a lysine residue of a substrate. In the case of catalytic E3 ligases, an additional trans-thiolation reaction occurs to transfer ubiquitin from the E2 catalytic cysteine to the E3 catalytic cysteine. The E3 then mediates covalent bond formation between the substrate and ubiquitin. **(B)** Ubiquitylation is a diverse modification, and distinct types of ubiquitylation – monoubiquitylation, multi-monoubiquitylation, and various polyubiquitylation linkages – are recognized by distinct ubiquitin-binding domains, leading to diverse final outcomes for the ubiquitylated protein.

Conjugation of ubiquitin to lysines on target proteins (substrates) occurs in a series of steps, illustrated in Figure 1A. First, ubiquitin is activated in an ATP-dependent manner by conjugation to an E1, or ubiquitin activating enzyme. Second, in a trans-thiolation reaction, ubiquitin is transferred from the E1 to the catalytic cysteine of an E2, or ubiquitin conjugating enzyme. The ubiquitin-conjugated E2 then interacts with an E3 ubiquitin ligase, to allow ubiquitylation on a lysine residue of a substrate (8, 12). E3 ubiquitin ligases bring substrate specificity to this reaction. There are two types of E3 ubiquitin ligases: those that have intrinsic catalytic activity, homologous to E6AP carboxyl terminus (HECT) type and really interesting new gene (RING)-between-RING (RBR) ligases, and those that do not, RING or U-box ligases (13). RING or U-box E3 ligases serve a scaffolding function, bringing ubiquitin-loaded E2 into close proximity to target protein lysines to facilitate covalent bond formation between the target lysine and the C-terminal glycine of ubiquitin. HECT-type ligases also bind to substrate and ubiquitin-loaded E2, but, in a second trans-thiolation reaction, ubiquitin is passed from the catalytic cysteine of the E2 to the catalytic cysteine of the E3, which then catalyzes ubiquitylation of the target lysine directly (14). RBR ligases have HECT-like catalytic activity in one RING domain, while the other retains the more common RING function, namely, the capacity to bind an E2 (15).

Ubiquitylation of a protein substrate can result in monoubiquitylation – the addition of a single ubiquitin to one or many accessible lysines of the substrates (known as multi-monoubiquitylation) – or can lead to the formation of polyubiquitin chains on the substrate (Figure 1B). These distinct post-translational modifications are recognized as unique signals by proteins containing ubiquitin-binding domains (16). Polyubiquitylation occurs when ubiquitin is processively conjugated to itself via one of its seven solvent accessible lysines (8, 9). It is worth noting that “head to tail” linked linear polyubiquitin chains, in which ubiquitin is linked via its N-terminal methionine were recently characterized; this unique linkage has been reviewed elsewhere (17).

Polyubiquitin chains linked via different lysines have distinct macromolecular structures. These distinct macromolecular structures are recognized by ubiquitin-binding proteins specific for certain polyubiquitin structures; these ubiquitin binding proteins ultimately direct distinct downstream protein fates. For example, K63 chains can provide a scaffold for protein complex formation. By contrast, “canonical” K48 chains are recruited to the proteasome, and thus promote degradation. However, distinct chains can target a protein for the same fate: all “atypical” ubiquitin linkages except K63 (e.g., lysine 6, 11, 27, 29, or 33) are recognized by proteasomal ubiquitin-binding domain proteins (18). The purpose of this redundancy is as yet unknown, and it is further complicated by the fact that more complex, branched chains of mixed linkages (heterotypic chains) also occur, although few downstream effects of these mixed linkages have been determined in cells (9, 19).

Ubiquitylation involves formation of covalent bonds; however, as with other post-translational modifications, ubiquitylation is reversible. Ubiquitin can be removed from proteins by deubiquitylating enzymes (DUBs), which can be specific for certain types of ubiquitin linkages and/or ubiquitylated protein substrates (12, 20). DUB activity is also required to generate a pool of free ubiquitin monomers from ubiquitin precursors, establishing these enzymes as key to initiating the ubiquitylation cascade (21). Thus, ubiquitylation is a highly dynamic, complex, and controlled form of molecular regulation, and a form of post-translational modification that impacts almost all proteins in the cell. To deal with such a vast array of possible targets, the substrate-specific components of the ubiquitylation cascade – E3 ligases and DUBs – show extensive diversity, while E1 and E2 enzymes are highly conserved and limited in number. Putative and verified ubiquitin enzymes in the mammalian genome are predicted to number more than 700 (12).

Our understanding of how ubiquitylation modulates TCR signaling is still in its infancy. Genetic loss-of-function studies are beginning to reveal some of the key enzymes and accessory proteins involved. Based on these studies, we now know that E3 ligases and DUBs affect many steps within the signaling cascades downstream of the TCR that culminate in the activation of critical transcription factors, such as NF-κB, NFAT, and AP-1. Several E3 ligases have been shown to be key players in the induction of anergy and in the differentiation of T helper cell subsets (10). However, for most E3 ligases and DUBs with described roles in the immune system, the specific substrates that regulate the impacted signaling pathways have yet to be identified. Indeed, for many E3 ubiquitin ligases and DUBs, whether substrate binding or enzymatic function is required for their observed function in T cells has yet to be directly tested.

The studies done to date identifying substrates and roles for E3 ligases and DUBs in T cells have uniformly been targeted in nature, based on use of genetic loss-of-function mouse or cell models. This approach has characterized many critical ubiquitylation events in activated T cells. However, these targeted studies are inherently limited in scope and fail to integrate previously described ubiquitylation events downstream of the TCR with newly defined ubiquitylation substrates. Thus, more global profiling of ubiquitylation events is needed to validate previous findings and uncover new roles for ubiquitylation in T cells in a high-throughput manner, enabling more targeted future studies. In this regard, ubiquitylation studies in immune cells lag behind phosphorylation studies. Global phosphoproteome studies have characterized dynamic phosphorylation events that occur during engagement of the TCR and co-stimulatory molecules (22–28). Similar studies have yet to be done for ubiquitylation in T cells. New quantitative proteomics platforms have recently been developed that have the potential to identify ubiquitylation events critical for T cell function, validate previous mechanistic findings, and reveal relationships between ubiquitylation and protein fate in a more global fashion.

In this review, we have focused on families of E3 ligases and DUBs that are known to regulate TCR signaling. The paucity of mechanistic information demonstrates the need for new tools, techniques, and further research to reveal how ubiquitylation regulates substrate fate and function and how this impacts TCR signaling events. To this end, we discuss the potential and practical limitations of new proteomic approaches to probe the ubiquitylome of T cells, and thus aid in unbiased screening of substrates.

### E3 Ubiquitin Ligases

E3 ubiquitin ligases have one common feature – the ability to select the protein substrates in the cell that will be covalently “tagged” with ubiquitin, thereby imparting substrate specificity to the ubiquitin cascade. Each E3 contains one or more domains that allow substrate binding, as well as a domain that binds an ubiquitin-conjugated E2. In the case of the HECT-type E3 ubiquitin ligases, E2 binding occurs within the HECT domain, while among RING type ligases E2 binding occurs via the RING domain. Currently, there are only a few catalytic HECT-type ligases with known functions in T cells – all of these are members of the neuronal precursor cell expressed and developmentally down-regulated protein 4 (Nedd4) family discussed below. All other E3 ubiquitin ligases with known roles in TCR signaling are non-catalytic, RING-type ligases. In Table 1, we have categorized several well-known E3 ubiquitin ligases, described in more detail below, based on the role of the ligase in promoting/preventing T cell activation.

**Table 1:** **E3 ligases can promote or inhibit activation of T cells**.

Promotes T cell activation and proliferation	Limits T cell activation	Ties T cell activation to co-stimulation	Induces T cell anergy
Nedd4	Itch	TRIM30	GRAIL
WWP2	Nedd4-2	TRAF6	Cbl-b
TRIM28	TRIM27	Cbl-b	Itch
Cul1	Peli1	Itch	
Stub1	Cbl-b		
TRAF6	(c-Cbl)		
TRAF2			

## Casitas B-Lineage Lymphoma Ligases

The three highly homologous casitas B-lineage lymphoma (Cbl) proteins, Cbl (c-Cbl, Cbl2, or RNF55), Cbl-b (also termed RNF56), and Cbl-c/Cbl-3 (also termed Cbl-SL or RNF57), have a RING domain, which allows interactions with E2 conjugating enzymes (31, 32), and multiple protein–protein interaction domains to facilitate their selection of substrates (33, 34). These protein–protein interaction regions include a tyrosine kinase-binding domain (TKB), a Src homology (SH2) domain, a proline-rich motif, an ubiquitin-associated (UBA) domain, and additional motifs known to be phosphorylated in a signal-dependent manner. Cbl-b and c-Cbl were among the first E3 ubiquitin ligases implicated in TCR signaling (29, 30). The third family member, Cbl-c, is not known to be expressed in T cells. Their diversity of interaction motifs make Cbl-b and c-Cbl particularly well suited for binding mediators of signaling cascades, such as those downstream of the TCR.

Cbl-b negatively regulates T cell activation. Mice lacking Cbl-b spontaneously develop autoimmune disease as they age and are more susceptible to induced forms of autoimmune disease. This is not due to a defect in the thymic selection of Cbl-b deficient T cells, as Cbl-b is predominantly expressed in T cells only after they have completed thymic development (35). Rather, T cells lacking Cbl-b can become fully activated in the absence of CD28 co-stimulation (29, 30, 36). Additionally, *in vitro* Cbl-b deficient CD4^+^ T cells show increased IL-2 production and proliferation in response to TCR/co-stimulation (29, 30). In peripheral T cells, TCR engagement drives activation of NFAT, which in turn leads to Cbl-b expression (37). Once expressed, Cbl-b has been proposed to mediate ubiquitylation of multiple TCR signaling mediators, including PLC-γ, the PI3 kinase subunit p85, and PKCθ (29, 30, 37–40). However, whether these are the relevant substrates remains somewhat controversial (41), and the precise means through which Cbl-b regulates TCR signaling via these and other substrates remains to be defined.

c-Cbl, like Cbl-b, negatively regulates TCR signaling. Unlike Cbl-b, c-Cbl is expressed predominantly in the thymus where it regulates levels of the TCR and signal strength upon receptor ligation. T cells lacking c-Cbl have enhanced Zap-70 phosphorylation, elevated TCR levels, and altered thymic selection (42, 43). Following TCR ligation, Zap-70 recruits c-Cbl to ubiquitylate the TCRζ chain (44). Interestingly, Zap-70-deficient thymocytes do not show defects in TCR surface expression (45, 46), supporting that other molecules, such as SLAP, may help recruit c-Cbl to the TCR complex (47–51). Once ubiquitylated, the TCR is degraded within lysosomes, as degradation is blocked by the use of lysosomal inhibitors (51) or deficiency in lysosomal-associated proteins, such as LAPTM5 (52, 53). Although c-Cbl has been shown to ubiquitylate other substrates, such as WASP (54), p85 (55), and CD5 (56), the relevance of ubiquitylation of these substrates in TCR signal modulation is less well-defined.

The similar yet non-redundant role of c-Cbl and Cbl-b in T cells is emphasized by the exacerbated phenotype of mice with doubly deficient T cells (57). Conditional deletion of both c-Cbl and Cbl-b in T cells leads to robust T cell-mediated inflammation mice: doubly deficient CD4^+^ T cells show defective surface TCR downregulation after ligand engagement, leading to prolonged signaling and T cell hyperesponsiveness (57).

More recently, Cbl-b has been described to work with other E3 ligases. Cbl-b can bind to the prototypic member of the Nedd4-family of E3 ubiquitin ligases, Nedd4 (58, 59). Nedd4 and Cbl-b have been shown to regulate each other’s function, either through degradation or by recruitment of the ligase to other factors (58, 59). Additionally, as described below, Cbl-b can work with STIP1 homology and U-box containing protein 1 (Stub1) to ubiquitylate FoxP3 (58–60).

## Neuronal Precursor Cell Expressed and Developmentally Down-Regulated Protein 4 Ligases

The neuronal precursor cell expressed and developmentally down-regulated protein 4 (Nedd4) family of catalytic HECT type E3 ubiquitin ligases is highly conserved, with an ortholog in budding yeast (61). These catalytic E3 ubiquitin ligases serve double duty in the ubiquitin cascade – providing both substrate specificity and catalyzing the final transfer of ubiquitin to accessible lysines on the target protein. As with other catalytic E3 ubiquitin ligases, Nedd4-family members are regulated by autoinhibition and activated by phosphorylation or through interactions with accessory proteins (62). The nine Nedd4-family proteins expressed in mammals share a modular architecture consisting of two to four WW domains that facilitate protein–protein interactions, a lipid and calcium-binding C2 domain, and the catalytic HECT domain. These nine Nedd4-family members constitute ~1/3 of the known HECT type E3 ligases in mice and humans (14, 63). Of the nine family members, evidence exists in the literature for expression of four of these in T cells: Nedd4, Nedd4-2 (or Nedd4L), WWP2, and Itch. The similarity of the Nedd4 family members has made mechanistic studies particularly challenging. In many cases, substrates identified for one family member are capable of being ubiquitylated by most other family members *in vitro*. Thus, while certain substrates have been described as being shared or context specific, cases of mistaken identity have also occurred (37, 62).

Nedd4 the archetypal family member, was originally characterized, along with the highly homologous Nedd4-2, as a negative regulator of epithelial sodium channel (ENAC) expression (64–67). Follow-up studies revealed numerous other shared, as well as unique, substrates for these E3s, including other ion channel and growth factor receptors (63). These substrates have not been specifically investigated in T cells, despite clear relevance for many of these proteins in T cell survival and TCR signaling. In the case of Nedd4, homozygous deletion resulted in embryonic lethality, and no T cell-specific knockout has been made; therefore, studies of Nedd4 function in T cells have been limited. Using fetal liver chimeras, Yang et al. found that Nedd4 promotes TCR signaling via degradation of Cbl-b (59). Due to accumulation of Cbl-b, T cells lacking Nedd4 were hypo-responsive to stimuli. An additional substrate of Nedd4, and perhaps WWP2 and Nedd4-2 as well (63, 68, 69), PTEN is also thought to have a role in *Nedd4*^−/−^ T cell hyporesponsiveness. Guo et al. proposed that, in the absence of Cbl-b, Nedd4 promotes PTEN inactivation via degradation, leading in part to the hyper-responsive phenotype of *Cbl-b*^−/−^ T cells (70). There is some evidence that Nedd4 can also act as a negative regulator of T cell responses, as, together with Itch, Nedd4 was proposed to degrade Bcl10 in order to limit NF-κB responses after TCR stimulation (71).

A thorough characterization of the role for Nedd4-2 in T cells is also lacking, as knockout mice show lethality at 3 weeks of age (72) and no T cell-specific knockout mouse has been made. In CD4^+^ T cells, however, Nedd4-2 was recently published to degrade JunB, a transcription factor previously published as a substrate of the Nedd4-family member Itch (73). However, this remains to be validated and described in more detail.

WWP2 is a much less studied family member, and to date no phenotype for knockout animals has been published. Using exogenous over-expression in T cells, one study identified WWP2 as a positive regulator of T cell activation, similar to Nedd4, as WWP2 was found to degrade Egr2, thereby limiting activation-induced cell death (74). The finding that PTEN can be degraded by WWP2 in certain transformed cell lines (69) suggests that WWP2 could regulate PTEN levels and/or function during T cell activation, although this has not been investigated.

Of the Nedd4 family members, Itch is the best studied in immune cells due to the striking immunological phenotype of Itchy mutant mice, which develop fulminant auto-inflammatory disease, characterized by Th2 skewing of CD4^+^ T cells (75, 76). Along with gene related to anergy in lymphocyte (GRAIL) and Cbl-b, Itch is considered to be a tolerogenic E3 ligase with important functions in restraining inappropriate immune responses. In activated T cells, Itch has been proposed to degrade PKCθ and PLCγ1 (37). In mouse peripheral T cells, one well-characterized substrate of Itch is JunB, an IL-4 transcription factor that accumulates in the absence of Itch, contributing to the T_H_2-type inflammation in Itchy mice (76, 77). However, over-expression of JunB only partially recapitulates the Itchy mouse phenotype, suggesting that other factors are also targets of Itch E3 ligase activity. Prior to the description of Itch as an E3 ligase for JunB, Itch was shown biochemically to degrade Notch. This may explain why Itchy mutant hematopoietic stem cells display increased proliferation and long-term self-renewing properties characterized by increased Notch1 signaling (78–81). FoxO1 was recently described as a target of Itch in vaccinia responsive T_FH_, but how FoxO1 is regulated in other T cell compartments has yet to be addressed (82). Itch also has direct relevance to human disease, as humans with mutations in Itch also show immunological defects (83). However, the molecular mechanisms within human immune cells leading to aberrant immune responses have not been described.

## Tripartite Motif Ligases

Tripartite motif protein (TRIM) E3 ligases are named for the presence of the tripartite motif, which contains a RING domain, one or two B-Box domains, and a coiled coil domain (84). This unique motif is invariably located at the N terminus, while there is broad heterogeneity in the C-terminal domains (85). TRIMs interact to generate homotypic and heterotypic multimers, forming discrete macromolecular structures in specific cellular compartments (84, 86, 87). TRIMs are a large, heterogeneous family in mammals, with ~70 proteins in both mice and humans. Analysis of the C-terminal domains suggests that TRIMs have diversified extensively in vertebrates, perhaps in response to pathogens (88, 89). The E3 ligase function of TRIMs is not limited to ubiquitin – although TRIMs can promote ubiquitylation through their interaction with E2 ubiquitin conjugating enzymes, TRIM RING domains can also mediate sumoylation and ISGylation with a variety of other E2s (90–92). Much work has focused on a role for TRIMs in pathogen responses, particularly in promoting antiviral responses in innate immune cells (93–103). This literature has been the subject of several reviews (104–107). However, little is known about the role of TRIMs in adaptive immune cells.

The most well-characterized TRIM in CD4^+^ T cells is TRIM28 (also called KAP/TIF1α), which contains histone interacting domains in addition to the tripartite motif, and is inducibly phosphorylated during TCR signaling (108). *TRIM28*^fl/fl^ CD4Cre^+^ mice have defective TCRα rearrangement and reduced peripheral T cell numbers; these TRIM28-deficient T cells have impaired cell proliferation and IL2 production (108, 109). Somewhat surprisingly, however, these mice develop autoimmune disease, characterized by high percentages of T_H_17 cells and defective Tregs (108).

Several other TRIM family members have known or suggested roles in CD4^+^ T cells. TRIM27 is a negative regulator of CD4^+^ T cell activation by promoting degradation of PI3KC2β, thereby limiting calcium release and preventing a sustained calcium signal after TCR engagement (110). Similarly, TRIM30 also appears to negatively regulate TCR signals, as CD4^+^ T cells from *TRIM30*^−/−^ mice show a loss of co-stimulatory molecule dependence and increased homeostatic proliferation upon transfer into *Rag1*^−/−^ recipients (111). Within innate cells, TRIM21 promotes ubiquitylation of IRF3, IRF7, and IRF8, with either pro- or anti-inflammatory effects on cytokine production (112–114). One study of *TRIM21*^−/−^ mice determined that several dysregulated cytokines are related to T_H_17 development (115); however, whether this is due to T cell intrinsic hyper-cytokine production remains to be seen. While many TRIMs have particularly high expression in innate cells, some are preferentially expressed within CD4^+^ T cells at the RNA level, including TRIM1, TRIM9, TRIM18, and TRIM46 (116). To date, no findings on these TRIMs in T cells have been published.

## Cullin Ring Ligases

The cullin RING ligases (CRLs) are the largest known family of E3 ligases. Translational research on cullins has primarily focused on their potential role in cancer because they are known to affect genes involved in cell cycle progression, cell proliferation, apoptosis, and DNA repair (117). Despite the relevance of these processes to T cell function, minimal work has been done on cullins in immune cells. Cullin proteins themselves do not possess an E3 ligase domain or function. Rather, cullins are the central scaffold of CRL complexes. CRLs are composed of cullin scaffold, a RING-box protein, a substrate recognition protein, and, in most cases, an adapter protein linking the cullin to the substrate recognition protein. There are 8 cullin family members, but there are over 200 CRLs due to the modular nature of these complexes (118, 119). The RING-box protein, Rbx1 or Rbx2, binds to the globular C-terminal domain (CTD) of the cullin and promotes CRL enzymatic activity by interaction with an E2 conjugating enzyme. The cullin CTD also contains a conserved lysine that must be NEDDylated in order for the cullin to be in its active form (117). Within the cullin N-terminal domain (NTD), cullin repeats either bind directly to a substrate-binding protein or indirectly via an adapter protein (119, 120). Assembly and structure of CRL complexes have been studied extensively and have been reviewed elsewhere (121).

The most well-studied member of the cullin family is CUL1. CUL1 expression in human tissues is highest in the spleen, blood, and tonsils, suggesting a possible immune function (122). CUL1 CRLs, also known as SCF complexes, are a complex of the adaptor Skp1, Rbx1, and 1 of nearly 70 different F-box proteins. The substrate specificity of the SCF complex is thought to be determined by the F-box protein (118). Of these 70 CUL1 CRLs, only 3 have been fully characterized in terms of substrate and function (118, 123). Substrates of the SCF are within known TCR signaling pathways and include the IFNα receptor 1 and IκBα (122, 124–126). However, only one study has specifically looked at the role of CUL1 in TCR signaling. In this study, TCR stimulation led to the ubiquitylation of p27^kip1^ by SCF^skp2^. p27^kip1^ ubiquitylation and subsequent degradation allowed T cells to proceed into S phase. TCR stimulation increases expression of Skp2, the F-box protein that targets the ligase activity of SCF to p27^kip1^, in contrast transcription of CUL1 and Skp1 was not affected (127).

Two other cullins have known roles in T cells, although their function in TCR signaling is unclear. Recently, it was shown that knockdown of CUL2 and CUL3 in a T cell line led to a robust increase in IL-2 production following TCR stimulation (10). CUL3 is the only member of the cullin family for which a T-cell-specific knockout mouse exists. Ratios of thymic CD4^+^ and CD8^+^ T cells are normal in these mice, but specific effector cell populations are disrupted. Levels of T_FH_ cells are abnormally high and NKT cells are significantly reduced in number (128). CUL3 associates with the BTB-ZF family of transcription factors to make epigenetic changes that direct the differentiation of these two T cell populations. CUL3 leads to the promotion of the NKT cell lineage and the inhibition of T_FH_ cells by associating with PLZF and Bcl6, respectively (128, 129). This phenotype was shown to be independent of TCR signaling (129), so it remains unclear whether and how CUL3 regulation of TCR signaling regulates T cell biology.

Perhaps due to the relevance of cullins in cancer, several unbiased proteomic screens have been developed in cell lines and successfully used to identify CRL substrates bound by different F-box proteins. These screens have utilized substrate-trapping mutations in the F-box proteins that prevent release of the ubiquitylated substrate, tagged versions of F-box proteins for specific immunoprecipitation, and the chemical inhibitor of the Nedd8-activating enzyme to prevent cullin activation (130–133). These large scale studies have achieved good specificity and reproducibility; however, utility of these published work flows is likely limited in primary immune cells due to the requirement for numerous replicates and a transfectable cell type.

## Gene Related to Anergy in Lymphocytes

As indicated by its name, gene related to anergy in lymphocytes (GRAIL) is a RING-type E3 ubiquitin ligase that is crucial for the induction of anergy in T cells. Resting T cells generally express low levels of GRAIL; these levels are further decreased following TCR/CD28 stimulation (134). Conversely, expression of GRAIL is rapidly increased following anergic stimulation of T cells. Resting T cells that over-express GRAIL produce greatly reduced IL-2 in response to TCR stimulation – this reduced IL-2 production depends on GRAIL E3 ligase activity (135). GRAIL over-expressing Jurkat and DO11.10 T cells exhibit impaired actin polarization at the immunological synapse following TCR stimulation, and also demonstrate impaired lymphocyte function-associated antigen (LFA) polarization and JNK phosphorylation downstream of TCR stimulation (136).

GRAIL knockout BALB/c mice have normal numbers and ratios of lymphocytes. However, established methods of inducing oral tolerance in these mice are ineffective (137). Aged GRAIL knockout mice develop autoimmune disease with infiltration of lymphocytes in their lungs and kidneys (138). CD4^+^ T cells from GRAIL knockout mice are more sensitive to TCR stimulation, exhibiting hyperproliferation and increased production of IL-2 and IFN-γ (137). The CD3ζ chains have been implicated as a possible target of GRAIL’s E3 ligase activity. Following stimulation with αCD3 alone, there are fewer TCRs on the cell surface and a concordant increase in ubiquitylation of CD3ζ; in the absence of GRAIL, this downregulation of the TCR does not occur (138).

## Pellino 1

The three Pellino family members are highly homologous, and all contain a RING domain that is critical to their function. In T cells, Pellino 1 (Peli1) acts as a negative regulator of T cell activation via its interaction with the NF-κB pathway. Cells deficient in Peli1 are hyper-responsive following TCR stimulation, with increased production of IL-2 and IFN-γ. These cells are also resistant to TGF-β-or Treg-mediated suppression (139). The hyper-responsive phenotype of Peli1 knockout cells correlates with increased protein levels of NF-κB and increased nuclear c-Rel following TCR stimulation. Peli1 has been shown to ubiquitylate c-Rel, forming K48 chains that initiate its degradation (139). Peli1 knockout mice have increased numbers of memory T cells in both the spleen and lymph nodes. Similar changes in T cell populations are seen among Peli-deficient T cells in mixed bone marrow chimeras, indicating that Peli1 acts intrinsically on these T cell populations. Aged Peli1 mice develop autoimmune disease with anti-nuclear antibodies in the serum, immune complexes in the kidneys, and lymphocyte infiltrates in the kidneys, liver, and lungs (139).

## STIP1 Homology and U-Box Containing Protein 1

The U-box E3 ubiquitin ligase Stub1, also known as carboxy terminus of HSP70-interacting protein (CHIP) plays an important role as a “co-chaperone,” associating with protein folding machinery to promote degradation of aberrantly folded proteins during new protein synthesis. Consistent with this role, Stub1 is highly expressed in metabolically active cells and tissues, in which a high level of translation is maintained (140, 141). In T cells, Stub1 has been shown to regulate NF-κB activation following TCR ligation, suggesting a role in tuning T cell activation. Stub1 also limits the levels of FoxP3 after TCR stimulation, supporting a role in Treg cell fate.

Specifically, Stub1 is thought to amplify NF-κB signaling through ubiquitylation of CARMA1. Jurkat T cells with Stub1 knocked-down via RNAi show decreased phosphorylation of IκBα, decreased ubiquitylation of CARMA1, and decreased transcription of IL-2 *in vitro* (142). Following TCR ligation of regulatory T cells, Stub1 has been shown to ubiquitylate FoxP3. In Jurkat T cells constitutively expressing FoxP3, degradation of FoxP3 following TCR and inflammatory cytokine signaling depends on Stub1 ubiquitylation of FoxP3. Accordingly, knockdown of Stub1 led to the accumulation of FoxP3 in this cell line (143). Stub1 also associates with FoxP3 upon TCR stimulation. Both Stub1 and Cbl-b can ubiquitylate FoxP3 following TCR stimulation, but Stub1 may play a more dominant role as T cells deficient in Cbl-b show reduced ubiquitylation, whereas T cells deficient in Stub1 have a complete lack of FoxP3 ubiquitylation (60). Much of the work thus far on Stub1 has been performed using cell lines or over-expression systems. Given that *Stub1*^−/−^ mice develop spontaneous atopic lung inflammation (144), while it remains unclear whether or how these mechanisms regulate T cell function *in vivo*, an intrinsic role for Stub1 in regulation of activated T cells seems likely.

While many E3 ligases and DUBs operate as part of cellular quality control mechanisms, as is the case for Stub1, few also have specific, separable roles in signal responses. Thus, it will be interesting to see if future research identifies the Stub1 targets described thus far as client proteins of molecular chaperones.

## TNF Receptor-Associated Factor Family

TNF receptor-associated factor (TRAF) proteins are defined by their association with membrane-bound receptors in the tumor necrosis factor receptor (TNFR) family. All TRAF proteins have a conserved TRAF domain at their C-terminus that interacts both with these associated membrane receptors and with other TRAF proteins to form hetero- and homodimers. All TRAF proteins except TRAF1 have an N-terminal RING domain (145). TRAF proteins facilitate K63 polyubiquitylation of their substrates, and thus promote protein–protein interactions as opposed to targeting the substrate for proteasomal degradation (146).

While multiple TRAF E3 ligases are expressed in T cells, the most well-characterized in regards to TCR signaling is TRAF6. TRAF6 is required for NF-κB pathway activation following TCR stimulation. Knockdown of TRAF6 in Jurkat T cells greatly reduces NF-κB activation, likely due to loss of IκBα phosphorylation. TRAF6 can ubiquitylate MALT1 oligomers via K63 linkages that promote protein–protein interactions (147). The ubiquitylation of MALT1 leads to the recruitment of NEMO/IKKγ to the CARMA1–MALT1–BCL10 (CMB) complex (148) where it is phosphorylated. TRAF6 also ubiquitylates NEMO (147) and recruits Caspase8 to lipid rafts. All of these steps are required for effective NF-κB signaling in T cells following TCR ligation (149). Somewhat surprisingly, although TRAF6 activates NF-κB *in vitro*, the primary phenotype of T cell-specific TRAF6 knockout mice is systemic autoimmune disease and a failure to induce anergy (150, 151). T cells from these mice are resistant to suppression by Treg cells and do not require CD28 co-stimulation for their activation. These cells also have constitutively activated Akt and enhanced phosphorylation of p85 following TCR stimulation (even without co-stimulation). This hyperactivation of PI3K pathway components may be responsible for the resistance to anergy in TRAF6-deficient T cells (150). Additionally, TRAF6-deficient T cells have reduced Cbl-b expression following anergizing conditions (151). Although the specific connection between TRAF6 and Cbl-b remains unknown, dysregulation of Cbl-b could also contribute to the loss of anergy in TRAF6-deficient T cells.

TRAF2 is also involved in the activation of the NF-κB pathway in T cells, but the underlying mechanism is poorly understood. TRAF2 acts on the non-canonical NF-κB pathway through a signaling complex termed the OX40 signalosome. TCR and OX40 signaling leads to an association between OX40 and protein kinase B (PKB), and PKB activation. This association is reduced in TRAF2-knockdown T cells, resulting in decreased PKB activation and reduced IL-2 expression (152), suggesting that TRAF2 ubiquitylation plays an activating role in formation of the OX40 signalsome. The OX40 signalosome can also activate the CMB complex leading to NF-κB activation. TRAF2-knockdown T cells fail to fully activate NF-κB likely due to defective formation of the signalosome complex (153).

Genetic loss-of-function studies for other TRAFs hint toward a role in T cell function, but mechanisms are broadly lacking. For example, TRAF1-deficient mice have increased numbers of lymphocytes in peripheral lymphoid compartments and an increased T/B cell ratio. T cells from these mice are hyper-proliferative in response to TCR stimulation, and/or when stimulated with IL-2 (154). By contrast, T cell-specific TRAF3 knockout mice show normal numbers of total T cells but increased numbers of Treg cells and CD4^+^ effector/memory T cells, with reduced numbers of CD8^+^ T cells (155, 156). *In vitro*, CD4^+^ T cells from these mice showed reduced phosphorylation of ERK, LAT, PLCγ1, and ZAP70, and reduced proliferation and cytokine production in response to TCR stimulation (155). This is quite different from what is seen in TRAF5 knockout mice. These mice show no obvious phenotype or immune deficiency, but when challenged using a model of airways hypersensitivity, they show increased T_H_2 lung inflammation compared to WT mice. *In vitro*, T cells from these mice preferentially differentiate into T_H_2 cells following activation of TCR/OX40 signaling (157).

### Deubiquitylating Enzymes

The deubiquitylating enzymes (DUBs) are a class of proteases that cleave ubiquitin from its target protein, thus allowing ubiquitylation to be a reversible process. At this time, 95 putative DUB genes have been reported within the human genome based on having one of the five ubiquitin-specific protease domains: ubiquitin-specific protease (USP), ubiquitin C-terminal hydrolase (UCH), ovarian tumor (OTU), Machado-Josephin (MJD), or JAB1/MPN/Mov34 metalloenzyme (JAMM) (158). These five domains define the five subclasses of DUBs. Most DUBs act as cysteine proteases, relying on a cysteine followed by a histidine in the catalytic site. The histidine allows for the deprotonation of the cysteine residue, which then cleaves the C-terminus of ubiquitin by nucleophilic attack (20, 158). The substrate of a DUB can be a specific ubiquitin polymer or a specific target protein, but targets (substrates) for most DUBs are currently unknown. Mass spectrometry of epitope-tagged proteins has identified factors that interact with DUBs and have elucidated potential roles for DUBs in specific biological processes, many of which are relevant in T cells (159).

## Ubiquitin-Specific Proteases

Ubiquitin-specific proteases (USPs) are one of the main families of cysteine proteases, and the largest family of DUBs. Since cleavage occurs between two ubiquitins within a polyubiquitin chain, most USPs contain at least two ubiquitin-binding sites, one for the distal ubiquitin and the other for the proximal ubiquitin (160). Several USP family DUBs have been shown to regulate TCR signaling, including USP9x, USP15, and USP7.

Usp9x is highly expressed in the spleen and thymus of mice (161–163), indicating a possible function in T cells. T cells from chimeric mice with shRNA knockdown of Usp9x demonstrate defective proliferation and reduced IFN-γ, IL-2, IL-4, and IL-17 production, indicating an inhibition of helper T cell differentiation or cytokine production (162). This inhibition seems to be due to a decrease in NF-κB activation following TCR stimulation in Usp9x knockdown T cells. Jurkat T cells with Usp9x knockdown show decreased phosphorylation of IκBα, decreased nuclear p65, decreased Bcl10 interaction with CARMA1, and increased ubiquitylation of Bcl10 (162). Thus, Usp9x may be acting on the NF-κB pathway via deubiquitylation of Bcl10. Specifically, Usp9x appears to deubiquitylate K48 ubiquitin linkages on Bcl10 following TCR stimulation (162). Experiments in mice with a conditional knockout of Usp9x in T cells contradict this finding. Although T cells lacking Usp9x have diminished proliferation following TCR stimulation, they show no decrease in nuclear translocation of p65 or in the interaction between Bcl10 and Carma1 (163). Instead, there is a decrease in phosphorylated LAT and PLC-γ1 following TCR stimulation. Mice lacking Usp9x in T cells ultimately develop a lupus-like autoimmune disease with splenomegaly and anti-nuclear antibodies (163). Usp9x has been shown to deubiquitylate Itch, protecting Itch from degradation in the proteasome after auto-ubiquitylation. Thus, increased degradation of Itch in the absence of USP9x could provide an alternative explanation for the increased autoimmunity in Usp9x-deficient mice. However, the experiments examining Itch have been carried out *in vitro* in HEK-293T cells and COS-7 cells, not in T cells (161).

Recently, two additional USP DUBs have been published as having roles in T cells. USP15 is abundantly expressed in T cells, and T cells lacking this enzyme show increased IL-2 and IFNγ production. USP15 deubiquitylates MDM2, an E3 ligase that ubiquitylates and degrades P53 as well as NFATc2. In the absence of USP15, MDM2 levels are decreased and NFATc2 levels in the nucleus consequently increase, likely accounting for elevated IL-2 and IFNγ production (164). Increased T cell responsiveness led to improved pathogen clearance as well as reduced tumor-induced lethality (164). USP7 decreased immune activation by binding to and stabilizing FoxP3 in regulatory T cells, perhaps serving to counteract the actions of Stub1 and Cbl-b, which can promote FoxP3 ubiquitylation (143, 165). Though USP7 and Stub1/Cbl-b have not been investigated together, this post-translation regulation of FoxP3 may be an example of ubiquitin editing. Undoubtedly, future work will uncover additional USP family members that play important roles in modulating TCR signaling and responsiveness.

### Ubiquitin Editing

The relationship between E3 ligases and DUBs parallels that of kinases and phosphatases. The antagonistic functions of E3 ligase and DUBs can quickly create and modify post-translational modifications, and the balance of their functions sets the signaling state of the cell. Ubiquitin, however, is not simply an on/off switch. The fate of the substrate depends not only on the presence or absence of ubiquitin but also on the length and specific linkage type of the attached ubiquitin chain. Ubiquitin itself has seven lysine residues, thus providing seven different locations for linkages in a polyubiquitin chain. The type of ubiquitin chain attached to a substrate dictates the fate of that protein. For example, while a K48-linked chain targets the substrate for proteasomal degradation, a K63-linked chain may recruit other signaling molecules to the substrate. Both E3 ligases and DUBs show preference for forming/removing certain types of ubiquitin linkages. For example, the TRAF E3 ligases (Traf2, 3, and 6) build K63 ubiquitin chains, whereas Usp9x removes K48 ubiquitin chains (162, 166). The number and diversity of E3 ligases and DUBs make ubiquitylation an incredibly dynamic post-translational modification. The dynamic alteration of ubiquitin chains is known as ubiquitin editing.

Because the precise substrates of many E3 ligases and DUBs are still unknown, the exact molecular interactions in most instances of ubiquitin editing remain elusive. Ubiquitin editing of NF-κB is the most well-studied model. The OTU-domain containing DUB, A20, plays a key role in the suppression of the NF-κB activation and uniquely demonstrates ubiquitin editing by contain both deubiqutylating and E3 ligase domains (167, 168). A20 deficient mice die within 3 weeks of birth due to rampant inflammation, likely due to over-activation of NF-κB (169). Because it contains both DUB and E3 ubiquitin ligase domains, A20 theoretically has the potential to form and edit chains on substrates without partnering with other factors. This might be the case in the interaction of A20 with one of its known substrates, the kinase RIP. Downstream of TNF receptor signaling, RIP activity promotes activation of NF-κB; this is terminated by A20. Mechanistically, A20 is thought to facilitate the removal of K63 ubiquitin chains, which promote activity of RIP, and add K48 ubiquitin chains, thus targeting RIP for proteasomal degradation (170).

Most research on A20 has focused on its role in innate immune cells, where its function as an ubiquitin editor is well established. Few studies have focused on the role of A20 in NF-κB signaling following TCR activation. In contrast to innate immune cells, TNF does not induce the expression of A20 in T cells. Instead, A20 is constitutively expressed in mature resting T cells but is immediately down-regulated following T cell activation (171, 172). In T cells, TRAF6 and A20 work in functional opposition to edit MALT1 ubiquitylation, thereby keeping NF-κB activation in check downstream of TCR stimulation. TRAF6 promotes K63 ubiquitylation of MALT1, leading to the association of the Carma1–MALT1-Bcl10 (CMB) complex with IKK, subsequent phosphorylation/activation of IKK, and NF-κB activation (148). It has been proposed that A20 facilitates deubiquitylation of MALT1 to limit the CBM–IKK interaction and suppress NF-κB activation (172). Thus, TRAF6 and A20 ubiquitin editing of MALT1 provides a rheostat for T cell activation.

Structure–function studies have recently challenged the direct role of A20 in ubiquitin editing. Specifically, A20’s role as a DUB was recently called into question. A knock-in mouse that specifically lacks A20 DUB activity was found to have normal NF-κB signaling and no overt phenotype (173), suggesting that A20’s DUB function is dispensable for normal NF-κB signaling, and its ubiquitin editing function may rely on its other domains (such as its E3 ligase RING domain) or higher complex formation. However, A20 may be required as a DUB in other signaling cascades. Specifically, A20 DUB activity was shown to regulate necroptosis in T cells, as well as in other cell types (174).

Similar to A20, the UCH-type deubiquitylating enzyme CYLD limits NF-κB activation by deubiquitylation. While A20 acts as a negative feedback mechanism to NF-κB stimulation, CYLD is thought to suppress basal NF-κB activation. In the absence of any robust stimulation of NF-κB (such as TCR engagement or cytokine signaling), CYLD deubiquitylates TRAF2, TRAF6, and NEMO, dampening NF-κB signaling by removing activating K63 chains formed by TRAF2/6 (175, 176). Additionally, in both macrophages and T cells, CYLD inhibits the ubiquitylation and subsequent activation of Tak1, a kinase that activates both IKK and JNK (177, 178). Therefore, CYLD is a key negative regulator of both the NF-κB and AP-1 pathways through negative regulation of upstream signaling intermediates.

**Figure 2:**
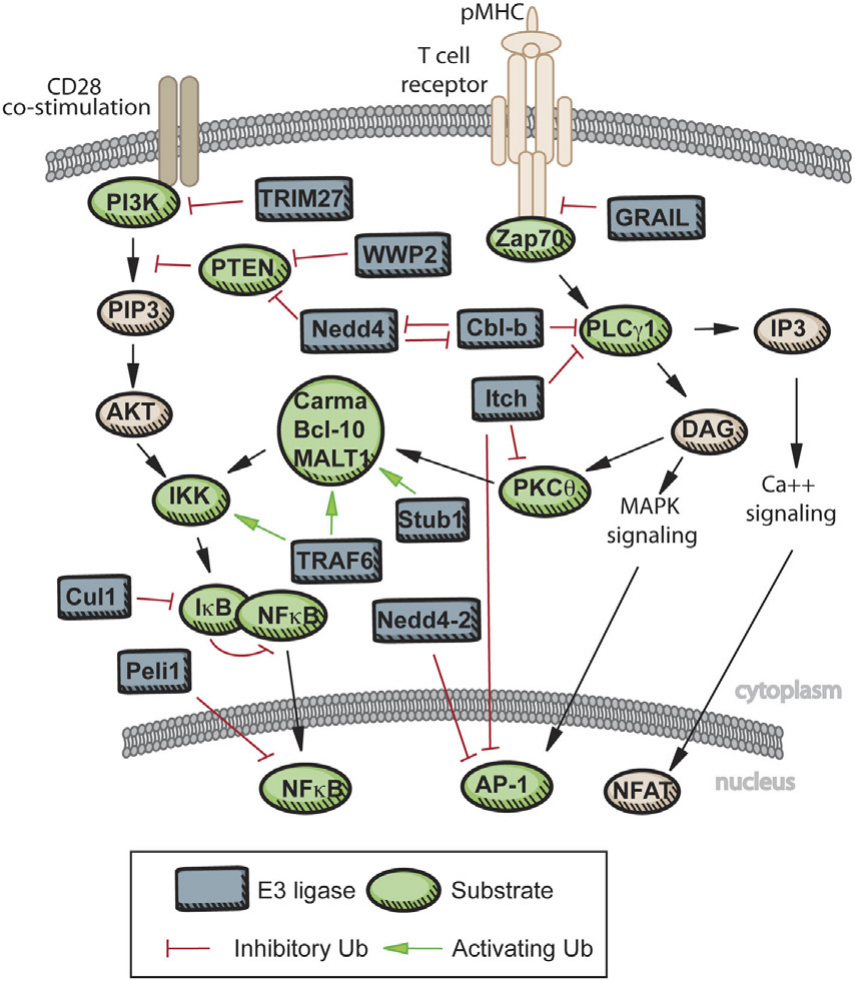
**Known roles of E3 ligase and DUBs in TCR signaling**. Upon engagement of the TCR with peptide-MHC, several E3 ligases act to limit the strength of TCR signaling. GRAIL, CBL-B, and Itch are active in limiting proximal TCR signaling, and therefore enforcing T cell tolerance. Additional E3 ligases are activated with engagement of co-stimulatory molecules, and these E3s can promote activation of a T cell receiving signals through both the TCR and CD28 (Nedd4, WWP2), or limit signaling downstream of CD28 (TRIM27). Further downstream, activation of NF-κB is dependent on K63 chain formation on a variety of scaffolding proteins, while activity of the AP-1 complex and nuclear localization of NFAT can be inhibited directly by E3 ligase activity or by repression further upstream.

CYLD and A20 demonstrate how the balance and specificity of ubiquitylation/deubiquitylation can create a dynamic system for modulating immune signaling. In Figure 2, we have summarized the known roles in T cells for the ligases described here. While limited evidence of ubiquitin editing exists to date in primary CD4^+^ T cells, the opportunity for interplay and co-regulation of certain substrates by E3 ligases and DUBs during TCR signaling is clear, providing an exciting opportunity for further defining how TCR signals are regulated, which may provide new targets for therapeutic modulation. In this regard, the controversy surrounding A20’s DUB function serves as an important warning that domain homology does not necessarily equate with function in all systems, and that detailed structure–function studies should be undertaken prior to attempts at therapeutic targeting. Figure 2 also highlights important aspects of many ubiquitylation studies in T cells: first, that targeted approaches typically yield only one enzyme–substrate relationship and, second, that several well-studied E3 ligases or DUBs have now been published with multiple, in some cases overlapping substrates. Thus, generally lacking from this work is integration of known ubiquitylation events with newly identified events into more cohesive signaling networks.

## Unbiased Proteomic Approaches for Identifying E3 Ligase Substrates in Primary T Cells

As exemplified in many of the specific ligase/DUB–substrate relationships described above, the post-translational attachment of ubiquitin to cellular proteins can alter their function and half-life in diverse ways, thereby profoundly impacting T cell signaling and function. Targeted approaches in T cells, such as knockout/knockdown or over-expression of E3 ubiquitin ligases/DUBs, have aided in our understanding of how ubiquitylation affects specific cellular processes, such as T cell activation or effector function, and identified many specific protein substrates targeted by the ubiquitylation cascade during T cell signaling. However, these targeted approaches are limited in scope, and many fail to identify any specific substrate of ubiquitylation that is affected in the absence of the E3 ligase or DUB of interest.

While many specific E3 ubiquitin ligase and DUBs have been identified that impact immune function, as our review of the literature indicates there are relatively few (if any) identified substrates of these ligases and DUBs. Indeed, the majority of known ubiquitylation targets in T cells are attributed to function of only a handful of well-studied E3 ligases in which there is a robust immune phenotype in loss-of-function mouse models. Furthermore, targeted studies of ubiquitylation enzymes typically describe a single substrate that is altered in the absence of the E3 ligase or DUB; in most cases, it is exceedingly unlikely that altered ubiquitylation status of a single protein would be sufficient to explain a complex immunological phenotype. In this regard, high-throughput analysis of changes in ubiquitylation of many potential targets would be exceedingly beneficial both in new substrate discovery and in validation of previous findings.

Genomic and transcriptomic analyses of T cells lacking these various proteins are likely to suggest only indirect effects, i.e., downstream of changes that result from alterations in a substrate protein’s levels or function. Proteomics, specifically the analysis of proteins using mass spectrometry, is the logical high-throughput method for studying post-translational modifications. Proteomic analysis of phosphorylation sites has been used with great success to catalog dynamic changes in phosphorylation that occur within minutes of T cell stimulation (22–28). Similarly, in the ubiquitylation field, proteomics has recently become de rigueur for global analysis of ubiquitylation sites and for substrate identification, although this has yet to translate to immunology.

**Figure 3:**
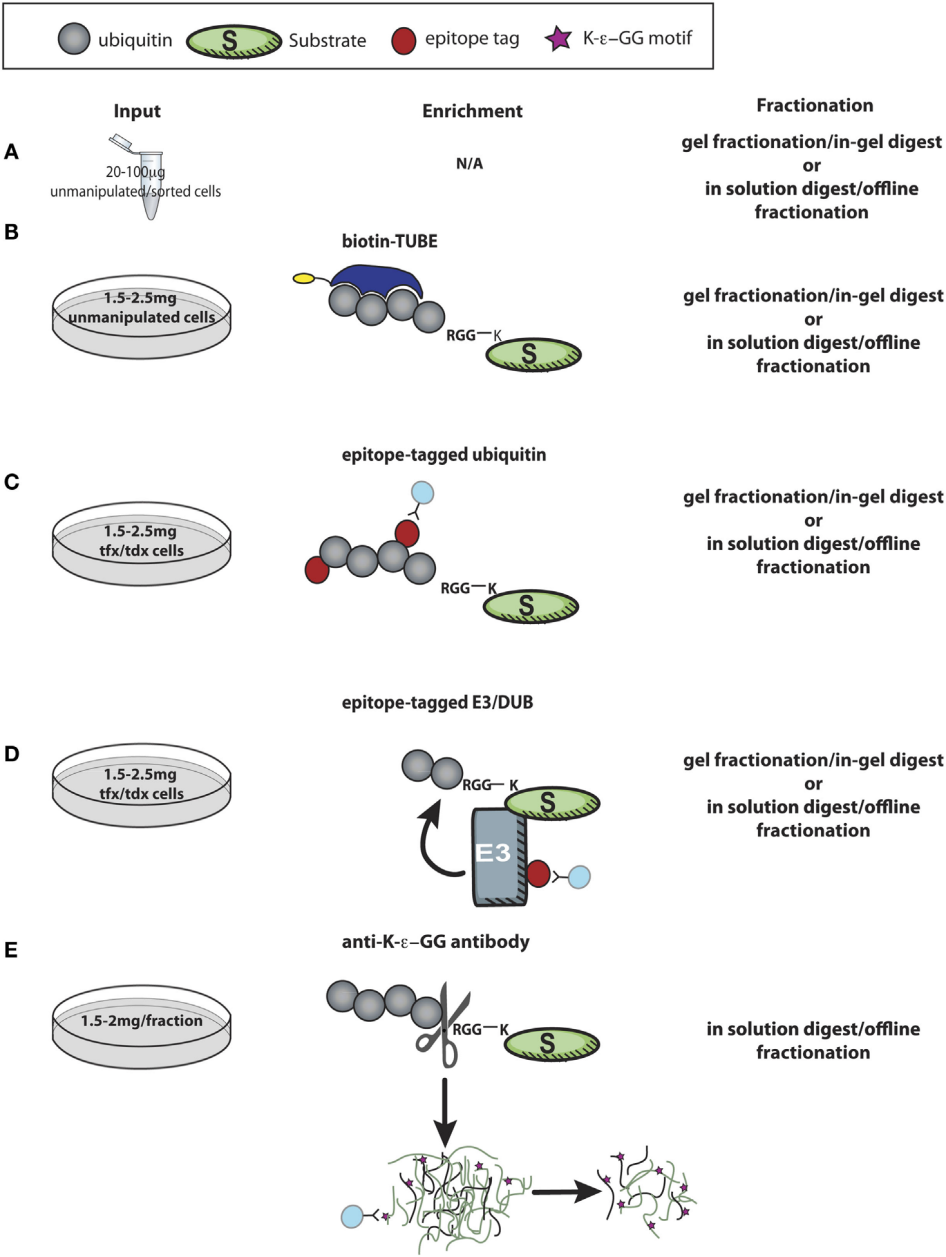
**Proteomic approaches to discover new targets of ubiquitin enzymes in T cells**. **(A)** Using minimal protein from recovered after lysing unmanipulated primary cells, LC-MS/MS analysis of control and experimental cells is useful to screen for relative expression of thousands of proteins, allowing further pathway analysis and combination with other datasets to identify possible targets of ubiquitylation. **(B,C)** Enrichment of polyubiquitin chains using a tandem ubiquitin-binding entities (TUBEs) by immunoprecipitation of epitope-tagged ubiquitin identifies thousands of proteins that are either directly ubiquitylated or involved in ubiquitin complexes with relatively minimal cellular manipulation, and ~1–2 mg of protein input. **(D)** More challenging in primary cells is expression of tagged E3 ligases or DUBs in primary cells to immunoprecipitate substrates, which may be bound only transiently and with low affinity to the enzyme of interest. **(E)** K-ϵ-GG peptide immunoprecipitation, perhaps the most direct proteomic approach to identify ubiquitylation, is challenging due to the large amount of starting material required (0.5–1 mg peptide/fraction). These approaches, and combinations there-of, hold great promise for future studies, both for hypothesis generation and investigations into molecular mechanisms of action for specific ubiquitylation enzymes. tfx = transfected, tdx = transduced.

Two general strategies have been used for high-throughput substrate identification. Candidate substrates can be identified based on observing interaction with the E3 ligase/DUB of interest, or a more global analysis can be performed to catalog thousands of ubiquitylated proteins, some of which may be changed in abundance in the absence or presence of a specific ubiquitylation enzyme. These are both mass spectrometry-based approaches that require high protein input. This presents a unique challenge for immunologists dealing with the limiting quantities of protein that can be obtained from primary lymphocytes. While limited studies have been done to date in primary T cells, ubiquitin-specific proteomic methods can be adapted for primary cells. In Figure 3, we have schematized several strategies, described below, for probing ubiquitylation in primary T cells via LC-MS/MS-based approaches.

The simplest proteomic approach to indirectly identify effects of perturbations in the ubiquitylation cascade is to compare levels of protein in experimental and control cells by LC-MS/MS analysis (Figure 3A). Such whole proteome analyses have been performed successfully on unmanipulated primary mouse and human cells (179–181), though not to probe explicitly for ubiquitylated proteins. This proteomic approach needs to be paired with control experiments to dissect transcriptional vs. post-transcriptional/translational effects. As ubiquitylation does more than lead to degradation of substrate proteins, altered protein abundance for specific substrates between experimental and control cells may not be observed. In this regard, reconstruction of a “virtual western blot” to identify polyubiquitin or ubiquitin-like chains on substrates by observing mass shifts in gel fractionated LC-MS/MS datasets may have some utility (182). Analysis of relative protein abundance is an important technique that is readily applicable to immune cells, as it requires little starting material to achieve good depth of proteome coverage and reproducible quantification of protein abundance. Although the detection of peptides from any given protein is dependent on the sample fractionation, the protein size, and trypsin (or other protease) cleavage sites, this type of relative abundance measurement can provide important insights into the relative expression of E3 ligases or DUBs as well as how abundance of these proteins are altered in distinct T cell subsets or under various T cell stimulation conditions.

A common approach to identify active ubiquitylation enzymes and their ubiquitylated substrates is to express epitope-tagged versions of ubiquitin (Figure 3C) or the enzyme of interest (Figure 3D). After immunoprecipitation against the tag, ubiquitylated proteins or proteins in complex are analyzed by LC-MS/MS to identify potential substrates that co-precipitated with the E3 ligase or ubiquitin itself (183, 184). If tagged ubiquitin is used, cell lysis and subsequent enrichment for ubiquitin can be carried out under harsh conditions, thereby minimizing detection of proteins that might not be directly modified by ubiquitin. For E3 ligase immunoprecipitations, which must be done under less stringent lysis conditions, identification of co-precipitating proteins can be coupled with *in vitro* ubiquitylation assays of bound proteins to identify putative substrates from non-substrate interaction partners. As substrate interactions may be fleeting, a more robust approach is to express a mutated “substrate-trapping” form of the ligase, which has been done successfully for SCF ligase complexes in cell lines (185). In most cases, this type of approach requires transfection/transduction, making it less feasible for primary immune cell applications, particularly in analysis of naïve cells. Utilization of CRISPR technology to engineer mice with epitope-tagged E3 ligases or ubiquitin may make these types of experiments more accessible for primary cell studies.

Alternative approaches exist for substrate screening based on modification by ubiquitin. In lieu of expressing tagged ubiquitin, enrichment of polyubiquitylated proteins from unmanipulated cells can be accomplished by using reagents containing linked ubiquitin-binding domains [such as tandem ubiquitin-binding entities (TUBEs), Figure 3B]. The linked ubiquitin-binding domains bind with high affinity to a variety of polyubiquitin chains, and can be used for affinity purification of endogenous polyubiquitylated proteins from unmanipulated cells (186–189). The ability to detect endogenous polyubiquitylated proteins via affinity purification makes ubiquitin-binding domains more attractive for use in primary cells. The use of reagents that bind polyubiquitin chains, as opposed to using tagged ubiquitin, can be beneficial if researchers do not want to detect monoubiquitylation events. However, as with the use of tagged enzymes for immunoprecipitation, with these reagents validation of direct ubiquitylation on candidate protein substrates is required, as enrichment is carried out under less stringent lysis conditions and likely to identify many cellular ubiquitin-binding proteins and protein members of larger ubiquitylation complexes in addition to direct substrates of ubiquitylation.

Ubiquitylation of a specific lysine can be directly observed by mass spectrometry. Lysines covalently modified by ubiquitin, or the ubiquitin-like proteins Nedd8 and ISG15 (UBLs), are protected from trypsin cleavage, and carry a diglycine, or ubiquitin remnant motif (K-ϵ-GG) after trypsin cleavage at the C-terminal arginine of ubiquitin, Nedd8, or ISG15 (183). These peptides are relatively rare in proteome analyses, even following enrichment on ubiquitin (~0.5–1% of proteins identified) (183). Development of an antibody against the ubiquitin remnant motif has revolutionized the ability to catalog ubiquitylated lysines. Direct immunoprecipitation of K-ϵ-GG peptides after enrichment on tagged ubiquitin significantly enriches the percent and number of ubiquitylated lysines and proteins identified compared to affinity purification of ubiquitin alone (190). The initial report using a monoclonal αK-ϵ-GG antibody characterized only several hundred ubiquitin remnant peptides in a similar number of proteins; however, this effectively doubled the known number of ubiquitin-modified lysines in the human proteome (190). Subsequent studies, also in cell lines, have significantly improved upon this. By omitting the tagged ubiquitin affinity purification step and using direct antibody enrichment of trypsinized proteins from unmanipulated cells (Figure 3E), several labs report 5000 to 20,000 ubiquitylation sites identified in several thousand proteins within a single experiment (191–193). This ubiquitin remnant “profiling” has made ubiquitylation the second most abundant post-translational modification annotated in the human proteome (second only to phosphorylation) (192). Furthermore, it has been used quantitatively to successfully identify substrates of E3 ligases (133, 191). Thus far, however, no one has reported use of ubiquitin remnant profiling in primary T cells. Large amounts of starting protein material are required for highly specific enrichment of ubiquitin peptides, representing a significant hurdle for those interested in applying this technique in T cells. However, the benefits of observing a protein with a modified lysine are clear. As an example of one advantage of this method, recently, it was reported that a lysine in RIPK3, discovered via ubiquitin remnant immunoprecipitation from control and *A20*^−/−^ MEFs, was required for *in vivo* RIPK3 ubiquitylation in T cells (174). Thus, characterization of ubiquitylated lysine residues has ready application for immune cell biology.

Demonstrating the power of combination approaches, recently it was shown that expression of TUBEs in cells protects effectively against DUB activity. Following direct affinity purification of the TUBE construct from cells, tryptic peptides were subjected to diglycine immunoprecipitation, and a significant increase in ubiquitin remnant peptides was observed thanks to the protection of a variety of ubiquitin chains from DUB action. This method was successfully used to identify substrates of a previously uncharacterized F-box protein (133). However, this method requires transfection of the ubiquitin-binding TUBE construct as well as the ligase of interest, and therefore will require significant optimization before achieving utility in primary T cells.

For all ubiquitin-based enrichment techniques, it is common to include proteasomal inhibitors, such as MG132, in cell culture prior to LC-MS/MS analysis. However, use of these inhibitors carries important caveats. First, during T cell stimulation, degradative events may be critical for additional activation signaling to occur. Second, use of these inhibitors creates ubiquitin-proteasomal stress, and, while it is predicted that only about 6% of all K-ϵ-GG peptides come from UBL linkages, use of inhibitors can lead to the depletion of free ubiquitin and an increased entry of UBLs into the ubiquitin conjugation cascade (191). Finally, in comparison to efficient protection against DUB activity, pharmacological inhibition of the proteasome may only incrementally increase the pool of ubiquitylated proteins in a cell, preferentially promoting a build-up of proteins with K48 polyubiquitylation (133).

Unlike the E3 ligases, where screens seek to identify substrates, unbiased screens for DUBs have relied primarily on activity profiling using chemical probes to identify isopeptidase activity of DUBs in cell lysates (194–199). Such probes can also be used to tag ubiquitin conjugating enzyme activity. Activity-based assays for E3 ligases utilize ubiquitylation *in vitro* to define new substrates. These assays are often dependent on recombinant expression of a range of possible substrates, and therefore more suited to specific hypothesis testing. Although there are commercially available protein microarrays suitable for discovery purposes, these currently exist in a large-scale format only for human proteins (200, 201). For both E3 ligases and DUBs, many of which are characterized as such by domain homology, activity profiling generates important data on the relevance of enzymatic activity in cells.

## Conclusion

Ubiquitylation is a post-translational modification with critical roles in immune cell homeostasis and function that are only now being elucidated. Far from leading only to the degradation of proteins as part of cellular “maintenance,” ubiquitylation of target substrates can have varied consequences on protein activity, localization, and half-life. The type of ubiquitin modification and also the availability of ubiquitin-binding proteins and DUBs, which cooperate with or antagonize E3 ubiquitin ligases, dictate these diverse consequences. In this review, we have described what is currently known about E3 ubiquitin ligases and DUBs that regulate TCR signaling and T cell biology. Where possible, we have indicated the molecular mechanism by which these proteins are thought to exert effects on TCR signaling, specifically in the activated CD4^+^ T cells. However, for the majority of these proteins, the molecular mechanism(s) by which they impact T cell signaling, or the immune system more broadly, remains unknown. Indeed, in some cases, it is still unclear whether the E3 ligase or DUB in question is acting via its ligase/DUB domains, as structure–function assays are largely lacking in T cells. We propose that proteomics techniques can be used in primary T cells to aid both hypothesis driven and exploratory experiments. Such studies will provide a more thorough understanding of how ubiquitylation pathways regulate TCR and other signaling pathways in CD4^+^ T cells and guide the rational design of therapeutics with which to treat immune-mediated diseases.

## Author Contributions

EL, PO, and CO wrote and edited this review.

## Conflict of Interest Statement

The authors declare that the research was conducted in the absence of any commercial or financial relationships that could be construed as a potential conflict of interest.
